# Space GlucoseControl system for blood glucose control in intensive care patients - a European multicentre observational study

**DOI:** 10.1186/s12871-016-0175-4

**Published:** 2016-01-22

**Authors:** Jan Blaha, Barbara Barteczko-Grajek, Pawel Berezowicz, Jiri Charvat, Jiri Chvojka, Teodoro Grau, Jonathan Holmgren, Ulrich Jaschinski, Petr Kopecky, Jan Manak, Mette Moehl, Jonathan Paddle, Marcello Pasculli, Johan Petersson, Sirak Petros, Danilo Radrizzani, Vinodkumar Singh, Joel Starkopf

**Affiliations:** 1 Department of Anaesthesiology and Intensive Medicine, 1st Faculty of Medicine, Charles University and General University Hospital Prague, U Nemocnice 2, 128 08, Prague 2, Czech Republic; 2 Department of Anaesthesiology and Intensive Therapy, Wroclaw Medical University, Wroclaw, Poland; 3 Department of Anaesthesiology and Intensive Care Medicine, Vejle Hospital, Vejle, Denmark; 4 Internal Medicine Clinic, University Hospital in Motol, Prague, Czech Republic; 5 Medical Department I, Faculty of Medicine in Pilsen, Charles University in Prague and University Hospital in Pilsen, Pilsen, Czech Republic; 6 Department of Anaesthesiology and Intensive Care Medicine, Capio Hospital Sur, Madrid, Spain; 7 Department of Anaesthesiology and Intensive Care Medicine, County Hospital Ryhov, Jönköping, Sweden; 8 Department of Anaesthesiology and Surgical Intensive Care Medicine, Klinikum Augsburg, Augsburg, Germany; 9 Department of Internal Medicine III – Metabolism and Gerontology, University Hospital Hradec Kralove, Hradec Kralove, Czech Republic; 10 Department of Cardiothoracic Anaesthesia and Intensive Care Unit, University Hospital, University of Copenhagen, Copenhagen, Denmark; 11 Intensive Care Department, Royal Cornwall Hospital, Truro, UK; 12 Department of Surgical and Intensive Medicine, Siena University Hospital, Siena, Italy; 13 Department of Anesthesiology and Intensive Care, Karolinska University Hospital Solna, Stockholm, Sweden; 14 Medical ICU, University Hospital Leipzig, Leipzig, Germany; 15 Department of Anesthesiology and Intensive Care, Legnano Hospital, Legnano, Italy; 16 Critical Care Services, Department of Anaesthetics, West Suffollk Hospital NHS Trust, Bury St Edmunds, UK; 17 Department of Anaesthesiology and Intensive Care, Tartu University Hospital, Tartu, Estonia

**Keywords:** Glycaemia control, Critically ill patients, Insulin protocol, Space Glucose Control, Enhanced Model Predictive Control (eMPC)

## Abstract

**Background:**

Glycaemia control (GC) remains an important therapeutic goal in critically ill patients. The enhanced Model Predictive Control (eMPC) algorithm, which models the behaviour of blood glucose (BG) and insulin sensitivity in individual ICU patients with variable blood samples, is an effective, clinically proven computer based protocol successfully tested at multiple institutions on medical and surgical patients with different nutritional protocols. eMPC has been integrated into the B.Braun Space GlucoseControl system (SGC), which allows direct data communication between pumps and microprocessor. The present study was undertaken to assess the clinical performance and safety of the SGC for glycaemia control in critically ill patients under routine conditions in different ICU settings and with various nutritional protocols.

**Methods:**

The study endpoints were the percentage of time the BG was within the target range 4.4 – 8.3 mmol.l^−1^, the frequency of hypoglycaemic episodes, adherence to the advice of the SGC and BG measurement intervals. BG was monitored, and insulin was given as a continuous infusion according to the advice of the SGC. Nutritional management (enteral, parenteral or both) was carried out at the discretion of each centre.

**Results:**

17 centres from 9 European countries included a total of 508 patients, the median study time was 2.9 (1.9-6.1) days. The median (IQR) time–in–target was 83.0 (68.7-93.1) % of time with the mean proposed measurement interval 2.0 ± 0.5 hours. 99.6 % of the SGC advices on insulin infusion rate were accepted by the user. Only 4 episodes (0.01 % of all BG measurements) of severe hypoglycaemia <2.2 mmol.l^−1^ in 4 patients occurred (0.8 %; 95 % CI 0.02-1.6 %).

**Conclusion:**

Under routine conditions and under different nutritional protocols the Space GlucoseControl system with integrated eMPC algorithm has exhibited its suitability for glycaemia control in critically ill patients.

**Trial registration:**

ClinicalTrials.gov NCT01523665

## Background

Glycaemia control (GC) remains an important therapeutic goal in critically ill patients, despite an ongoing debate regarding the optimum target ranges. The crucial question is the appropriate intensity of glycaemic control that can be accomplished safely. Risk of hypoglycaemia is the most important concern in GC implementation; therefore a protocolized approach, comprising a validated insulin administration protocol and use of accurate monitoring technologies is essential for safe GC management in intensive care patients. Standardizing GC by a nurse-managed protocol has been found to improve safety and efficiency [1]. In most intensive care units (ICU), protocols for GC are still paper-based flow-charts, using an “if-then” decision model [2, 3]. As increasing complexity of insulin protocols reduces their clarity for the user and increases the difficulty of their use, paper-based protocols became converted into computerized forms – from simple electronic algorithms to clinical decision support systems (CDSS) with the ability to predict insulin requirements based on patient-specific response to previous doses as a function of their individual insulin resistance pattern. In addition to improved GC, a computerized CDSS for GC has the potential to simplify the work for nurses, reduce overall workload and, most importantly, reduce the chance of human error [4–7]. Although comparison of existing protocols is difficult due to differences in processes and outcome measures, CDSSs generally achieve better GC with consistently lower hypoglycaemia rates than paper-based systems and appear to be superior in ICU patients [8–10].

The enhanced Model Predictive Control algorithm (eMPC) developed by the CLINICIP (Closed Loop Insulin Infusion in Critically Ill Patients) group [11] features a variable sampling interval based on the accuracy of the blood glucose (BG) prediction and is among those most clinically proven and effective both in medical and surgical ICU patients [10–12]. Being one of only a few predictive algorithms it has been successfully implemented in multiple institutions with different nutritional protocols and on different ICU populations, including paediatric patients.

The eMPC, as with other computerized algorithms, has to be used on bedside computers, limiting its use. Therefore the clinical application of the eMPC algorithm, the B.Braun Space GlucoseControl system (SGC), combines an effective algorithm with the advantages of direct interconnection of all entry data - blood glucose level, insulin rate and nutrition. SGC consists of three infusion pumps (two for nutrition, one for insulin), connected to a common station that allows data communication between the pumps and a central user interface (Space Control). Space Control and the associated SGC Module with the incorporated eMPC algorithm provide suggestions for insulin rate and glucose measurement interval.

Although SGC is already used in many ICUs worldwide, there are few published experiences with the use of the SGC system [13, 14], but none with its routine use.

Even for the recently published study by Amrein et al., performed at two tertiary academic centres (Medical University of Graz, Austria and the Medical University of Zurich, Switzerland), all actively participating nurses had to first attended a structured training before enrolment of the first patient to familiarize with the handling of the complete SGC system [15]. Therefore the objective of this multicentre observational European study was to describe the routine clinical performance and safety of the SGC for GC in critically ill patients in different ICU settings and also to test its ability to cope with various nutritional protocols, enteral, parenteral or combined. To ensure truly routine conditions, only those centres where the SGC were already routinely used, participated in this non-interventional study.

## Methods

### Study design

We conducted an observational multi-centre European study to evaluate BG control in adult intensive care patients using the SGC system. This system incorporates the eMPC algorithm to advise insulin titration for optimal GC.

The primary objective of the study was to assess the performance and efficiency of the SGC system for GC in ICU patients under routine conditions. Secondary objectives were the safety and usability of the SGC system. The primary study endpoint was the percentage of time within the target range (4.4 – 8.3 mmol.l^−1^), from the start of the treatment to the last glucose measurement under treatment. Initially two target ranges, 4.4-6.1 mmol.l^−1^ and 4.4 – 8.3 mmol.l^−1^, were set for the study, but based on the preference of each participating ICU only the wider range (4.4 – 8.3 mmol.l^−1^) was finally used within the study. Secondary outcome measures were frequency of hypoglycaemic episodes for safety evaluation, and adherence to the advice of the SGC, BG measurement intervals and technical parameters of the SGC (number of BG measurements outside the predicted range generating a plausibility warning and number of overruled insulin rate advices) for the SGC system usability assessment.

The study was performed in accordance with the guidelines proposed in the Declaration of Helsinki and was separately and independently approved by ethics committees of all participating institutions: Etická komise VFN v Praze*/General University Hospital Prague, Czech Republic; Etická komise FN Motol*/University Hospital in Motol, Prague, Czech Republic; Etická komise FN Plzeň*/University Hospital in Plzen, Czech Republic; CEIC Hospital ClínicoSan Carlos*/Capio Hospital Sur, Madrid, Spain; Ethik-Kommission der Bayerischen Landesartztekammer*/Klinikum Augsburg, Germany; Etická komise FN Hradec Kralove*/University Hospital Hradec Kralove, Czech Republic; Comitato Etico Azienda Ospedaliera Universitaria Senese*/Azienda Ospedaliera Universitaria Senese, Siena, Italy; Ethik-Kommission an der Medizinischen Fakultat der Universitat Leipzig*/Universität Leipzig, Germany; Comitato Etico Azienda Ospedaliera Ospedale Civile Di Legnano*/Azienda Ospedaliera di Legnano, Italy; Bioethics Committee of Wroclaw Medical University/Wroclaw Medical University, Poland; De Videnskabsetiske Komiteer for Region Hovedstaden/Vejle Sygehus, Denmark; Den videnskabsetiske komite i Region Hovedstaden/Rigshospitalet, University of Copenhagen, Denmark; South-West - Cornwall and Plymouth Research Ethics Committee/Royal Cornwall Hospital, Truro, UK; Etikkommitten Karolinska Universitetssjukhuset/Karolinska University Hospital Solna, Stockholm, Sweden; Research Operational & Governance Committee (ROC) on behalf of the Trust/West Suffolk Hospital NHS Trust, Bury St Edmunds, UK; Research Ethics Committee of the University of Tartu/Tartu University Hospital, Estonia.

Written informed consent, obtained before inclusion, or delayed consent from each patient or from a legal surrogate, was requested by local ethics committees in 9 centres (marked by an asterisk), while in remaining 8 centres consent was waived because of the observational nature of the study.

The study has been registered on January 26, 2012 at the Clinical Trials Database (ClinicalTrials.gov Identifier: NCT01523665).

### Study population

Eligible patients were adult ICU patients who required BG control by intravenous administration of insulin. Exclusion criteria were all contraindications to intravenous insulin therapy, decided by attending physicians. Only those data that were obtained in routine clinical use were recorded in the patient documentation sheet. No additional measures (visits, tests, investigations) were required for this non-interventional study.

Observation time per patient was started with initiation of the GC therapy using the SGC, i.e. the first BG measurement entered into the device, and ended when the GC therapy using the SGC system was finished. The initiation of GC depended solely on the discretion of the standard protocol of each participating ICU and was independent of the specific blood glucose value. Therefore in a number of centres GC was initiated also in patients without the presence of hyperglycaemia. The decisive criterion for inclusion of the patient was the availability of SGC at the time of GC initiation.

### Sample size, countries and sites

The study was intended to be performed in 20 sites in 9 European countries to cover different local clinical practices and was planned to include 1000 patients or be terminated on June 30, 2013 at the latest. Intensive care departments where the SGC was already in use were asked for participation. The number of patients included in each centre thus also depended on the number of SGC systems in use in each ICU.

### Space GlucoseControl

The Space GlucoseControl system is a decision support system for optimized insulin therapy for critically ill patients in a closely monitored environment, such as ICUs or operating theatres. The system is based on the Space pump platform. The SpaceControl touch screen, serving as a central user interface, is connected to the SGC module containing the self-learning eMPC algorithm. It calculates the appropriate insulin rates by considering the latest BG values together with the actual rates of enteral and parenteral nutrition. In addition, it determines the time to next BG sample, based on the accuracy of the blood glucose prediction. The SGC user can neglect the decision as suggested by the SGC and/or at any time decide to take an additional BG measurement. Within the study the system was used in routine clinical practice and according to its intended purpose and the instructions for use. All other aspects of patient care, including nutritional management, were carried out at the discretion of the treating clinicians. After the study termination the SGC users were asked to fill anonymously a questionnaire related to efficiency of glycaemia control, safety and workload.

### Insulin administration

Insulin was given by a central venous catheter as a continuous infusion according to the suggestions of the SGC. The medical staff always had the final decision to accept the suggested insulin rate or not. The system does not automatically change the rate of insulin on its own without the involvement of a human being, requiring a crosscheck by medical staff.

### Blood glucose ranges, time in target range

The target range used within the study was 4.4 – 8.3 mmol.l^−1^. Severe hypoglycaemia was defined as blood glucose of <2.2 mmol.l^−1^, the glycaemic range between 2.2 and 4.3 mmol.l^−1^ is reported as hypoglycaemia. Hyperglycaemia was defined as blood glucose above 8.3 mmol.l^−1^.

In order to obtain the percentage of time in each range, the BG values were linearly interpolated. The percentage of time in relevant range was calculated from linearly interpolated BG values for each patient separately by dividing the time in respective range with the total study time for the patient (from the start of the treatment to the last glucose measurement under treatment), and then multiplying by 100.

### Blood sampling

Blood glucose was monitored according to the suggestions of the SGC with variable sample interval. Blood samples for glucose measurement were obtained by means of arterial catheters whenever possible or central venous catheters; the use of capillary samples was discouraged but not forbidden. BG levels were measured with the use of blood gas or point-of-care analysers or central laboratory analysers based on routine use at each centre.

### Data documentation

With the first BG measurement that was entered into the SGC system, all entered BG values, the insulin infusion rate, the enteral/parenteral carbohydrate feeding rates of the pumps connected to the system and the suggestions by the eMPC algorithm were automatically recorded in the system log-files. After the SGC therapy of a patient was finished, the therapy log file of the SGC was exported by the local study coordinator at each institution via SpaceCOM on an USB data storage device and then via a secured web-based study interface into the study database.

The following parameters were recorded at the start of the SGC therapy: patient demographics (age, sex, height, and weight), APACHE II score, history of diabetes, admission diagnosis and concomitant diseases. After the SGC therapy of a patient was finished, source of blood for glucose measurements, BG measurement device, adverse device effects, reason for overruling the advice of the SGC and reasons for discontinuation of SGC therapy were manually entered in a web-based observational documentation sheet for each patient. The data that had to be entered manually were checked for completeness and plausibility. Finally completed web-based patient documentation sheets and the corresponding SGC log-file were validated by the data manager. If necessary, data clarification requests were sent to the investigator.

### Statistical analysis

All patients who received intravenous insulin using the advice of the SGC at least once and had a validated SGC log-file with completed documentation were included in the analysis. Unevenly distributed continuous variables are presented as medians and interquartile ranges (IQR), and normally distributed data as means ± standard deviation (SD). Categorical data are given as a number ( %). The percentage of time within the predefined glucose target range (4.4-8.3 mmol/l) was defined as primary endpoint for the assessment of SGC performance and efficiency. Frequency of hypoglycaemic episodes was set as secondary endpoint for safety evaluation; BG measurement intervals and acceptance of SGC advices on insulin infusion rate for system usability assessment. The following standard procedures for the comparison of subgroups were used: Fisher’s exact test for binary data, chi-squared test for kx2 tables (k > 2), U test according to Wilcoxon-Mann–Whitney, t-test for metric data, ANOVA (analysis of variance). All statistical tests were performed two-tailed with the pre-specified significance level of α = 5 %. An interim analysis to check the protocol safety (frequency of hypoglycaemic episodes) was performed after 300 patients. Data analysis was performed using SPSS® version 19 (SPSS Inc., Chicago, IL).

## Results

### Study participants

17 centres from 9 European countries included overall 508 patients with complete study documentation. The first patient was included on June 27, 2011 and the last patient finished on July 26, 2013. The patients’ demographic characteristics, history of diabetes, APACHE II score, admission diagnosis and entry BG are shown in Table 1. There were no significant differences between the study centres in anthropometrical data (age, gender, height, weight and BMI of the patients), but the centres differed significantly in main admission diagnosis, number of diabetic patients (32 - 78 %; median 46 %), and entry BG levels (3.4 - 40 mmo/l; median 9.3 mmo/l).

**Table 1:** Patients baseline characteristics and admission diagnosis

Patients characteristics (*N* = 508)	
Female	198 (39.0)
Male	310 (61.0)
Age [years]	65.6 ± 12.9
Height [m]	1.70 ± 0.1
Weight [kg]	85.1 ± 20.5
BMI [kg/m^2^]	29.0 ± 6.5
BMI above 25 kg/m^2^	375 (73.8)
BMI above 30 kg/m^2^	184 (36.2)
APACHE II [points]	21.1 ± 7.4
BG level at study entry [mmol/l]	10.6 ± 5.6
*History of Diabetes*	
Total	280 (55.1)
Type 1	33 (6.5)
Type 2	244 (48.0)
Other	3 (0.6)
Admission diagnosis	
Heart insufficiency	152 (29.9)
Respiratory insufficiency	104 (20.5)
Sepsis, septic shock	63 (12.4)
Gastrointestinal disease, bleeding	38 (7.5)
Diabetes, diabetic ketoacidosis	21 (4.1)
Acute pancreatitis	21 (4.1)
Cerebral hematoma, bleeding, infarction	19 (3.7)
Myocardial infarction	14 (2.8)
Vascular diseases	11 (2.2)
Renal insufficiency	11 (2.2)
Liver diseases	10 (2.0)
Infections	8 (1.6)
Meningitis/ Encephalitis	6 (1.2)
Other	30 (5.9)

Data are presented as mean ± standard deviation or number (%). APACHE II = Acute Physiology and Chronic Health Evaluation II Score, BMI = body mass index

### Blood glucose measurement

Blood samples for glucose measurement were obtained by means of arterial catheters (61 %) and central venous catheters (27 %) or as capillary samples (12 %). BG levels were measured with point-of-care (58 %) or blood gas (42 %) analyzers. Central laboratory analyzer was used in one patient only.

### Blood glucose Level

All study patients were assigned to the target range of 4.4 - 8.3 mmol.l^−1^. The GC characteristics are shown in Table 2. 300 (59 %) patients started with BG level higher than 8.3 mmol.l^−1^, while 204 (40.2 %) had BG level within target range at the start of insulin therapy. In 4 patients the SGC was initialized when BG level was below 4.4 mmol.l^−1^. In patients with entry BG level higher than 8.3 mmol.l^−1^ the median time to target range was 4.5 hours (IQR 2.3-8.3), in 7 (2.3 %) patients the target BG was not achieved during the study period. The total study time varied from 2 hours to 54 days.

**Table 2:** Blood glucose control characteristics

Blood glucose control characteristics	Number	
Total study time [days]	508	2.9 (1.9–6.1)
Blood glucose level at entry [mmol.l^−1^]	508	9.3 (6.8–12.3)
Time to reach target range [hours]	293	4.5 (2.3–8.3)
Mean insulin infusion rate [U/h]	508	3.3 (2.3–5.1)
*Whole study period*		
Mean blood glucose level [mmol.l^−1^]	508	6.9 (6.6–7.7)
Severe hypoglycaemia <2.2 mmol.l^−^ [Number of patients/number of episodes]	508	4 (0.8)/4 (0.01)
Hypoglycaemia (2.2–4.3 mmol.l^−1^) [% of time]	271	0.2 (0–1.4)
Target range [% of time]	508	83.0 (68.7–93.1)
Hyperglycaemia >8.3 mmol.l^−1^ [% of time]	485	14.7 (6.1–29.5)
*After reaching target range*		
Mean blood glucose level [mmol.l^−1^]	501	6.9 (6.6–7.2)
Severe hypoglycaemia <2.2 mmol.l^−1^ [Number of patients/number of episodes]	508	4 (0.8)/4 (0.01)
Hypoglycaemia (2.2–4.3 mmol.l^−1^) [% of time]	269	0.2 (0.0–1.4)
Target range [% of time]	501	88.2 (77.5–95.1)
Hyperglycaemia >8.3 mmol.l^−1^ [% of time]	437	10.5 (3.4–20.4)

Data are presented as median (IQR) or number (%)

**Fig. 1:**
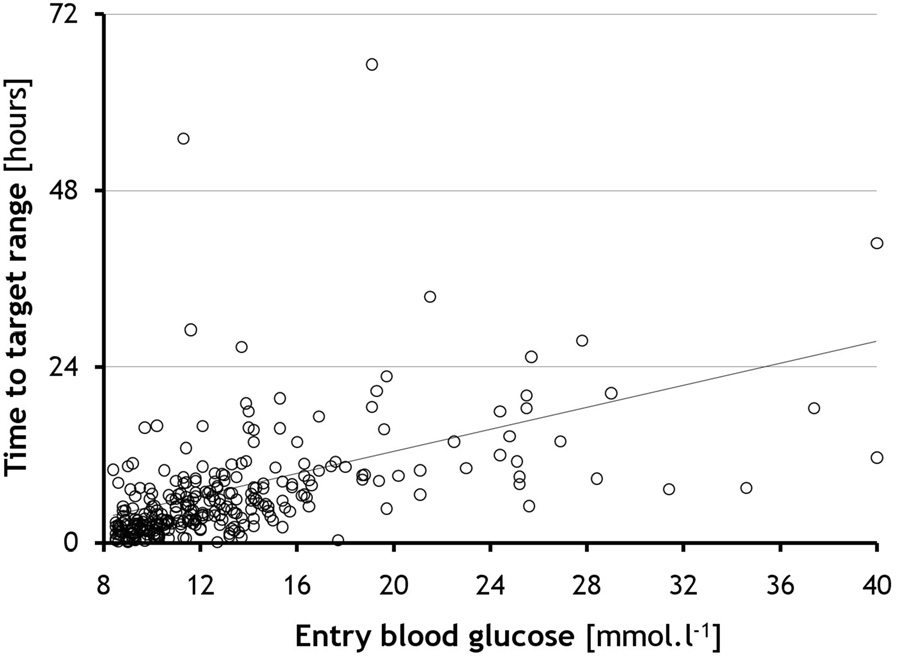
Correlation between entry blood glucose level and time to reach the target range. (*Pearson Correlation)*

**Fig. 2:**
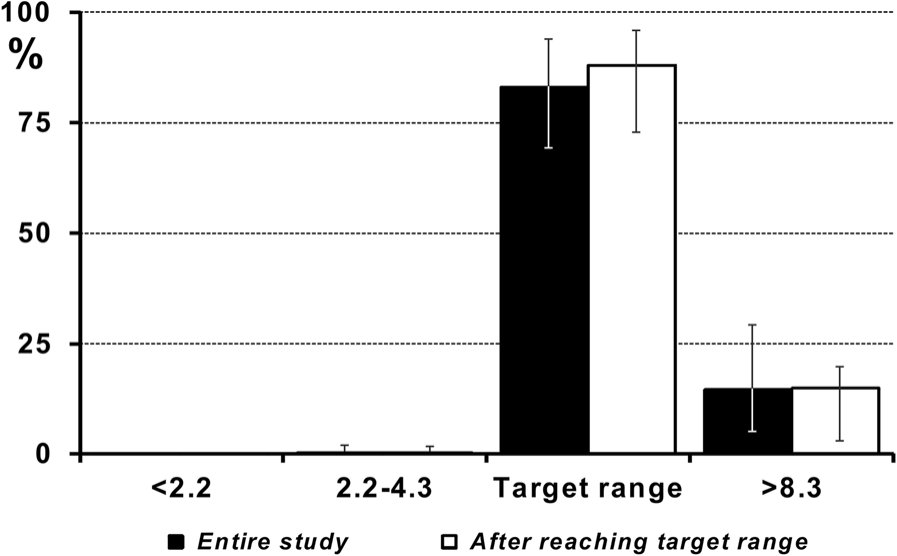
Time in target range. Time in each blood glucose range (mmol/l) for the entire study period (*black*) and from the first time in target range (*white*). *Data are presented as median (IQR)*

There was significant correlation (p < 0.001) between the entry BG level and the time to reach target range (Fig. 1). Good performance and safety of the system was observed with respect to both time in target range and incidence of hypoglycaemic episodes - only 4 severe hypoglycaemias in 4 patients (0.8 %; 95 % CI 0.02-1.6 %), representing 0.14 events/1000 measurements, were recorded in the entire study (Fig. 2).

### Technical parameters of the SGC system performance

A total of 29575 blood samples were drawn and corresponding number of recommendations for time to next sample were rendered by the SGC system during the whole study period. Mean proposed next measurement time was 2.0 ± 0.5 hours of which 0.15 % was shorter than 30 min, 46.6 % in the range 30–90 min, 22.7 % in the range 91–150 min, 12.3 % in the range 151–210 min and 18.4 % was longer than 210 min.

From the total of 29575 entered BG values 1176 (4 %) were found by SGC as not plausible (outside the range of the predicted BG level) and the SGC displayed an on-screen warning. 591 (50 %) of these warnings were because of unexpectedly too low, and 585 (50 %) because of unexpectedly too high BG values.

Based on BG values and nutrition changes the SGC gave a total of 30332 insulin infusion rate advisories. 118 of these were not accepted by the user – in 75 cases the insulin rate was adjusted downwards and in 43 cases upwards compared to the recommendations of the SGC system.

### Nutrition

Nutritional management was carried out at the discretion of the each participating centre and treating clinicians. The amount of carbohydrates given to the study patients is shown in Table 3.

**Table 3:** Overview on enteral and parenteral nutrition

Nutrition (carbohydrates)	
*Enteral nutrition*	
Enteral bolus [g] (*N* = 61; 12.0 %)	1.3 (0.5–57)
Enteral infusion [g/kg/day] (*N* = 287; 56.2 %)	2.1 (1.4–2.8)
Time with enteral infusion [% of total study time]	81.2 (58.7–94.7)
*Parenteral nutrition*	
Parenteral bolus [g] (*N* = 77; 15.2 %)	4.0 (0.5–10)
Parenteral infusion [g/kg/day] (*N* = 340; 66.9 %)	1.8 (1.2–2.7)
Time with parenteral infusion [% of total study time]	89.0 (60.9–97.3)
*Total nutrition*	
Enteral/parenteral [g/hour] (*N* = 464; 91.3 %)	7.2 (4.7–9.6)

Data are presented as median (IQR)

348 patients (68.5 %) received enteral nutrition; 287 as an infusion and 61 as a bolus at any time of the study. Parenteral carbohydrates were prescribed to 417 patients; in 340 cases as continuous infusions and in 77 as boluses.

### Users’ opinion

The SGC users (ICU nurses) were anonymously questioned after study termination about their opinion on the use of the SGC. Questions were related to efficiency of GC, safety and workload. The questionnaire was completed by a total of 170 ICU nurses. The answers to particular questions are shown in Table 4.

**Table 4:** Questionnaire. SGC users were questioned after the termination of the study (*N* = 170)

How well was the patient’s blood glucose stabilised in the target range?	Excellent	Good	Fair	Adequate	Poor
	15 %	61 %	16 %	6 %	2 %
Do you think that control of the patient’s blood glucose was maintained more effectively by the use of the SGC system compared with normal practice?				Yes	No
				74 %	26 %
Is the SGC system user friendly?				83 %	17 %
Did you feel confident using the SGC system?				81 %	19 %
Do you think that using the SGC system can help to avoid making mistakes when controlling the blood glucose?				77 %	23 %
How did you rate your work load using the SGC system compared with normal practise?	Decreased	Slightly decreased	Equal	Slightly increased	Increased
	1 %	2 %	6 %	31 %	60 %

Data are presented as %

*SGC* Space GlucoseControl

## Discussion

The objective of the present observational study was to assess the performance of the Space GlucoseControl system under routine clinical conditions in different European ICUs with different nutritional protocols. We observed very good performance of the system - 83 % of the time BG was maintained in the target range, and after reaching the target range even in 88 % of the time. On the other hand in 7 (2.3 %) patients the target BG range was not ever achieved during the study period. The incidence of hypoglycaemias was very low (0.2 %), demonstrating a good safety level. For comparison, a computer-assisted protocol used in the NICE-SUGAR trial, based on a complex decision tree had a hypoglycaemia rate of 6.8 % [16, 17].

Although SGC is already used in many ICUs worldwide, there are only few shared experiences with the use of this system [13–15]. In addition, the published data come from feasibility or testing studies. It is known that the results of such studies are often more optimistic than the results achieved under routine clinical conditions. Therefore the safety and effectiveness under clinical rather than study conditions are fundamental for safe GC management. Risk of hypoglycaemia is one of the most discussed issues in this field of critical care. In our study hypoglycaemia (<2.2 mmol.l^−1^) occurred in only 4 patients (0.8 %), representing 0.01 % of the time. Compared to the large clinical trials (Leuven studies, VISEP and NICE-SUGAR), where hypoglycaemia was frequent (from 5.1 up to 17 %)[16, 18–20], this is extremely low. From this perspective, the results of our study perhaps exceeded expectations. If we compare the achieved time in the target range in this study with the study by Amrein et al. performed in Graz and Zurich [15], the performance of the SGC under truly routine clinical conditions was exceptional as the time in target range 4.4 to 8.3 mmol/l was reached in both studies in 88 %. The same applies for the occurrence of moderate or severe hypoglycaemia.

Beside the predictive eMPC algorithm a key advantage of SGC is the direct interconnection of the algorithm with the standard infusion pumps - insulin and both enteral and parenteral nutrition. As one of the most common causes of severe hypoglycaemia in the ICU is a reduction in, or switching off of nutrition without proper adjustment of the rate of insulin [21], this connection is crucial to safety. The incidence of hypoglycaemic events with SGC is the lowest incidence of severe hypoglycaemia of all comparable studies, including those with eMPC algorithm [3, 10, 11, 14], albeit allowing for a narrower target range (4.4 – 6.1 mmol.l^−1^) in these trials. The comparison of existing insulin protocols is difficult, as the overall clinical settings including target ranges vary considerably between the studies. Therefore, the findings of such comparisons are rather speculative. For comparison with the SGC come into consideration mainly three other CDSS: the Glucommander - a web-based insulin nomogram, the GRIP (Glucose Regulation for Intensive care Patients) - a stand-alone Java based program, and EndoTool Glucose Management System - a predictive and adaptive software system for critical care setting. The Glucommander was invented in 1984 and is used in a number of mainly US and Canada hospitals [22]. In a study by Yamashita et al. the Glucommander maintained BG within the target range of 5.1–8.0 mmol/l for 53 % of the time and with hypoglycaemia occurred in 3.7 % from all BG measurements [23]. The performance of the GRIP seemed to be better, but at the first sight only. The fraction of time with BG between 4 and 7.5 mmol/l was 78 %, but with hypoglycaemia (<3.5 mmol/l) in 11 % of patients [24]! Similarly, with the EndoTool the percentage of BG measurements in the target range 4.4–8.3 mmol/l was 70.4 % and they experienced 7 hypoglycaemic events in 141 patients (vs. 4 events/508 patients in our study) [25]. Compare to these results the performance of the SGC in our study has shown somewhat superior efficiency with time in target range 83 % and also the safety with hypoglycaemia only 0.2 % of all measurements. Previous may also apply for other potential head-to-head comparisons with latest insulin protocol studies with similar target range: Software-guided insulin dosing protocol used by Saur and colleagues maintained glycaemia 68 % of time in the target range 5.3 -7.5 mmol/l with hypoglycaemia of 0.5 % [26]. SPRINT (Specialized Relative Insulin and Nutrition Tables) protocol, used at Hungarian ICU, achieved 77.6 % of BG measurements in the target range 4.0-8.0 mmol/l with 1.9 % of the BG <4.0 mmol/l [27]. And finally, with GlucoCare-directed Yale 100–140 mg/dL protocol targeted to 5.6-7.8 mmol/l the incidence of hypoglycaemia was 1.1 % of readings and 17.6 % of patients [28]. Comparison with other CDSS is rather impossible as their input conditions, measures and target ranges already differ too much.

An important verification of the SGC effectiveness and safety was its use under different ICU settings and with various nutritional protocols. As the intention of this study was to test the SGC under routine conditions, the feeding of patients was not standardized and nutritional management was carried out at the discretion of each participating centre. Hence a number of different nutritional protocols were used within the study. Regardless of the nutrition practices, the SGC algorithm coped safely with all of these variations. This versatility is achieved using a model of the glucoregulatory system within the eMPC, which adapts itself to the input–output relationship observed during GC. Incoming glucose measurements are used by the model to update model parameters such as insulin sensitivity, taking into account previously given insulin and parenteral and enteral carbohydrates. Once individualized to a critically ill subject, the eMPC uses the glucoregulatory model to determine the optimum insulin infusion rate to achieve the target glucose concentration.

The risk of human error is an important safety issue. Medication errors occur frequently with both paper-based and computerized protocols and play a major part in overall patient safety. In a retrospective study by Campion et al. over 5 % of BG values entered into the protocol did not match values from the analyser [29]. Other investigators have also reported rates of nurse protocol override, or deviation from system recommendations, ranging from 2 % to 23 % [29–32]. It is obvious that mismatched BG values can significantly influence protocol performance, both paper-based and computerized. From this perspective another advantage of the SGC is the prediction of the next BG level. The eMPC uses a model of the glucoregulatory system to predict a value of the next glucose measurement, taking into account individual insulin sensitivity and nutrition. If the entered value considerably differs from the predicted, the SGC evaluates this as not plausible and displays on-screen warning. In our study 4 % of entered BG values were found by the SGC as not plausible and the SGC asked for confirmation of the entered value, eventually recommending repeat measurement. Half of these warnings were because of unexpectedly too low BG, and half because of unexpectedly too high BG. A false hypoglycaemia would "only" risk exposing the patient to hyperglycaemia, however, a false hyperglycaemia exposes the patient to a risk of potentially severe hypoglycaemia as a result of increased insulin rate.

The acceptance of a therapy protocol by the critical care team (nurses as well as physicians) is a prerequisite for its successful implementation. Although this issue was not addressed in this study, data from the literature dealing with strategies of change management confirm this [33]. The complexity of critical care recommends therapeutic interventions that are easy to perform with a high level of safety and reproducibility. A computer assisted approach can fulfil these criteria. A recent published study comparing a paper-based with software driven protocol confirms the superiority of a computer assisted protocol in terms of sustained GC. However, the frequency of BG measurement increased nearly two-fold [26]. In our study the average interval between measurements was 2.02 hours. About 4 % of measurements were not plausible, resulting in the device recommending a repeat measurement and increasing the nurse workload. Although the implementation of SGC was on most ICUs associated with an increased workload for nurses, compared to their normal practice they evaluated improved effectiveness with the SGC highly positively, and mainly reported increased safety with respect to making mistakes when controlling the blood glucose in their patients. As the median stay in the study was only 2.9 days, frequent measurements might have been due to an unstable metabolism and insulin sensitivity in the early phase of critical illness. Further investigation of this device in patients with a long stay in the ICU is needed.

A limitation of our study is the use of different technologies for BG monitoring, including point-of-care, blood gas and central laboratory analyzers. However, the study was a pragmatic one designed to test SGC under routine conditions and this just emulates the routine practices in the ICUs and confirms the SGC performance in routine conditions. The same applies for the sampling sites for the measurement of BG. A potential future application of this system will be its connection to continuous glycaemic sensors. Feasibility studies have already shown a good performance of the eMPC algorithm in such an application [34].

## Conclusions

The Space GlucoseControl system with integrated eMPC algorithm has under routine conditions exhibited its suitability for blood glucose control in critically ill medical and surgical patients. Similarly, very low incidence of hypoglycaemias (0.2 %), acceptance of various nutritional protocols and only 0.4 % of recommendations not accepted by the user demonstrate its usefulness for routine blood glucose management under different ICU settings. It remains to be elucidated which cohort of patients (medical or surgical, type of surgery, type of nutrition, duration of stay, etc.) would benefit most from the application of the SGC.

## Appendix

### Participating investigators

Michal Porizka, Martin Stritesky, Lenka Vankova (*Department of Anaesthesiology and Intensive Medicine, General University Hospital Prague, Czech Republic*);

Andrzej Kübler, Barbara Dragan, Barbara Adamik (*Wroclaw Medical University, Dept. of Anaesthesiology and Intensive Therapy, Wroclaw, Poland*);

Anette Gammelgaard, Sabine Egebjerg (*Vejle Sygehus, Department of Anaesthesiology and Intensive Care Medicine, Vejle, Denmark*);

Katerina Lisova (*Faculty Hospital Motol Prague, Czech Republic*);

Martin Matejovic (*ICU, Medical Department I, Faculty of Medicine in Plzen, Charles University in Prague and Teaching Hospital Plzen, Czech Republic*);

Riccardo Giudici, Matteo Lucchelli, Giada Donà (*Dipartimento Anestesia e Rianimazione, Azienda Ospedaliera di Legnano, Legnano, Italy*);

Ilse Kreuzer (*Klinik für Anästhesiologie und Operative Intensivmedizin, Klinikum Augsburg, Augsburg, Germany*);

Finn Moeller Pedersen, Christine Helledie Olsen, Ingrid Greve (*Department of Cardiothoracic Anaesthesia and Intensive Care Unit, Rigshospitalet, University of Copenhagen, Denmark*);

Karen Burt (*Royal Cornwall Hospital, Intensive Care Department Truro, UK*);

Susanna Fantozzi, Maria Letizia Nucci (*Department of Surgical and Intensive Medicine, Azienda Ospedaliera Universitaria Senese, Siena, Italy*);

Eva-Maria Wallin, Ola Friman, Eva Engström (*Department of Anaesthesiology, Surgical Services and Intensive Care, Karolinska University Hospital Solna, Stockholm, Sweden*);

Sally Humphreys (*West Suffolk Hospital; Critical Care Services, Bury St Edmunds, UK*);

Silver Sarapuu, Kadri Tamme (*Tartu University Hospital, Department of Anaesthesiology and Intensive Care, Tartu, Estonia*)

## Abbreviations

APACHE II: acute physiology and chronic health evaluation II score
BG: blood glucose
CDSS: clinical decision support system
CLINICIP: closed loop insulin infusion in critically Ill patients
GC: glycaemia control
GRIP: glucose regulation for intensive care patients
eMPC: enhanced model predictive control
ICU: intensive care unit
SGC: Space GlucoseControl system
SPRINT: specialized relative insulin and nutrition tables
